# Metrics for Outpatient Portal Use Based on Log File Analysis: Algorithm Development

**DOI:** 10.2196/16849

**Published:** 2020-06-12

**Authors:** Gennaro Di Tosto, Ann Scheck McAlearney, Naleef Fareed, Timothy R Huerta

**Affiliations:** 1 CATALYST: Center for the Advancement of Team Science, Analytics, and Systems Thinking in Health Services and Implementation Science Research College of Medicine The Ohio State University Columbus, OH United States; 2 Department of Biomedical Informatics College of Medicine The Ohio State University Columbus, OH United States; 3 Department of Family Medicine College of Medicine The Ohio State University Columbus, OH United States; 4 Division of Health Services Management and Policy College of Public Health The Ohio State University Columbus, OH United States

**Keywords:** patient portals, health records, personal, health information technology, electronic health record

## Abstract

**Background:**

Web-based outpatient portals help patients engage in the management of their health by allowing them to access their medical information, schedule appointments, track their medications, and communicate with their physicians and care team members. Initial studies have shown that portal adoption positively affects health outcomes; however, early studies typically relied on survey data. Using data from health portal applications, we conducted systematic assessments of patients’ use of an outpatient portal to examine how patients engage with the tool.

**Objective:**

This study aimed to document the functionality of an outpatient portal in the context of outpatient care by mining portal usage data and to provide insights into how patients use this tool.

**Methods:**

Using audit log files from the outpatient portal associated with the electronic health record system implemented at a large multihospital academic medical center, we investigated the behavioral traces of a study population of 2607 patients who used the portal between July 2015 and February 2019. Patient portal use was defined as having an active account and having accessed any portal function more than once during the study time frame.

**Results:**

Through our analysis of audit log file data of the number and type of user interactions, we developed a taxonomy of functions and actions and computed analytic metrics, including frequency and comprehensiveness of use. We additionally documented the computational steps required to diagnose artifactual data and arrive at valid usage metrics. Of the 2607 patients in our sample, 2511 were active users of the patients portal where the median number of sessions was 94 (IQR 207). Function use was comprehensive at the patient level, while each session was instead limited to the use of one specific function. Only 17.45% (78,787/451,762) of the sessions were linked to activities involving more than one portal function.

**Conclusions:**

In discussing the full methodological choices made in our analysis, we hope to promote the replicability of our study at other institutions and contribute to the establishment of best practices that can facilitate the adoption of behavioral metrics that enable the measurement of patient engagement based on the outpatient portal use.

## Introduction

### Background

Patient portals are Web-based platforms administered by health care service providers that enable patients to access data held in their personal health record. Meaningful Use (MU), the US federal program that sought the promulgation of electronic health records (EHRs), and its associated incentives resulted in the widespread adoption of patient portals [1].

A notable component of MU required health care organizations to focus on improving patient engagement, specifically through the use of patient portals. This requirement came with the challenge of providing access to vulnerable members of the population (eg, ethnic and racial minorities, persons with disabilities, and those with lower health literacy skills), who are part of groups with higher medical needs in addition to facing technology access issues [2-4]. At the same time research has shown that investments in Web-based portals by health care organizations are increasing in order to meet demands from the general population, as patients want to use these tools at rates faster than they are made available [5]. As a result, many organizations now use outpatient portals that allow patients to access and view their prescriptions and medical information, schedule appointments, and communicate with their doctors [6].

Much of the research assessing the benefits of patient portals has focused on outpatient portals [7]. Studies have shown, for example, that outpatient portal users experience improvements in areas such as reducing risk factors for chronic diseases [8-12] and improving health outcomes [13,14]. However, what characterizes these prior studies is the generalized use of survey data for analysis as opposed to considering direct measures of online actions as a measure of engagement.

One approach to measuring engagement is based on the mining of EHR metadata. Modern EHR systems log every action taken by individuals signed into their systems. These actions create transactional traces on the EHR server in the form of log files, which can be leveraged as the foundation for analysis. Recent studies have made use of log file analysis as a means of assessing and understanding how and why patients engage in their care [7,15,16].

However, barriers remain to using this approach, including difficulty in tracking and accessing the data, the need to process a large volume of information, and the challenge of establishing use metrics. To lower such barriers to adopting this methodology, we add to the existing literature by documenting and describing the data wrangling process and its implications to enable future researchers to apply and validate our approach. To that end, together with our analysis, we publish the Stata code (StataCorp, 2017. Stata Statistical Software: Release 15, StataCorp LLC) required to replicate our analysis to contribute to the creation of shared data models that could enable the quantification of patient portal use behavior across portal types.

### Objectives

This methods-focused paper consequently addresses two aims. First, the study aimed to significantly expand the knowledge base of the methodological choices and challenges faced when processing log files. We provide documentation of our approach to process log files using data from our academic health system at the Ohio State University Wexner Medical Center (OSUWMC). This health system offers its patients electronic access to their health care information through the MyChart outpatient portal (Epic Systems Corporation) and has been in use since 2011. We intend to promote a standardized approach to this type of research by including our statistical program files as Multimedia Appendices 1-8 that can be used to analyze log files from Epic Systems. We do so by providing guidance to ensure that future studies adhere to the highest quality of data analysis when using audit log files of outpatient portal use. This approach is similar to the approach undertaken by Huerta et al [17], where a data model and procedure for processing log files from an inpatient portal were provided. However, given the differences between the outpatient portal and the inpatient portal log files, these data are idiosyncratic, affecting how they can be parsed; we note many of these differences in our Discussion section. We address this first study aim below in our Methods section.

The second aim of our study was to provide prescriptive data about outpatient portal usage to demonstrate the implications of our methodological approach and the assumptions about the decisions we made. Our results provide a glimpse into the outpatient portal usage in the context of the care provided at our institution. MyChart portal features include messaging with providers, prescription renewals, appointment information and scheduling, clinical updates, and billing. This approach could be replicated at other institutions that also desire to quantify outpatient portal use for different purposes such as quality improvement (eg, investigate tool performance), increasing patient engagement (eg, identifying the types of users), or for research (eg, summative evaluations of tool uptake). We address this secondary study aim in our Results section, in which we use descriptive statistics to present the portal use data.

## Methods

### Data Model

This section describes the data model used to examine outpatient portal use. It also documents the data sources required to produce the final data model and lists the recommended computational steps to clean and process the information extracted from the audit log files. The log files audited belong to a sample of 2607 OSUWMC patients, whose patient portal metadata from July 2015 to February 2019 were mined for the purpose of developing the methods to enable the study of patient engagement with Web-based portals. 

The analysis of outpatient portal use started with the categorization of all the individual actions performed on the portal by the patients. Actions were then aggregated across functions and over time to quantify user engagement with the technology. The resulting data model is represented in Table 1 and comprises four levels, with each subsequent level comprising the elements of the preceding level.

**Table 1:** Data model for outpatient portal use.

Data aggregation level	Definition
Action type	Single action performed by the user on the outpatient portal
Portal function	Category grouping user actions under the different functionalities offered through the outpatient portal
Session	Coherent, limited, and uninterrupted use of one or more of the outpatient portal functions via a sequence of user actions
Patient	Use of the outpatient portal, or one of its functions, across the entirety of a patient’s recorded sessions

Outpatient portal usage metrics were defined along the following two dimensions:

- Frequency of use: number of times an outpatient portal function has been accessed by a patient
- Comprehensiveness of use: number of unique outpatient portal functions accessed by a patient

Both dimensions were studied at the session and the patient levels of our data model.

### Data Sources

We audited the log files from MyChart’s instance at our institution to assess portal use. Data from these files included the date and time of the specific actions patients made using the outpatient portal (eg, appointment scheduling and viewing test results).

To support reproducibility of results, we document in Table 2 the sources for the data pertaining to the users’ MyChart actions and the status of their accounts by listing the exact variables and tables queried from Epic’s Clarity database.

Outpatient portal logs are recorded by Epic in the form of time-stamped sequences of user actions, and an example of this log is reproduced in Table 3. This table contains a sample from the MYC_PT_USER_ACCSS Clarity table, with personal identifiable information redacted. The first three columns present identifiers for a patient’s action, which include the medical record number (MRN), a time stamp, and a categorical variable indicating the type of action performed by the patient.

**Table 2:** Variables queried on the Epic’s Clarity database tables, to retrieve data about the users’ access to MyChart and their account status history, identified by their master table number by Epic System.

Variable name		Variable description	Master table number
**Patient information^a^**			
	PAT_MRN_ID	Patient identifier	EPT 2061
**Account status history log variables^b^**			
	MYC_STATUS_HX	Update to account status	EPT 28100
	MYC_STATUS_TMSTP	Status update time stamp	EPT 28110
	MYC_STATUS_MTHD	Technology implementing the update	EPT 28140
	MYC_STATUS_CMT	Additional status update description	EPT 28130
**User access log variables^c^**			
	UA_TIME	Action time stamp	WPR 520
	MYC_UA_TYPE_C	Action type	WPR 530
	UA_EXTENDED_INFO	Additional action type information	WPR 550
	UA_SESSION_NUM	Unique session identifier	WPR 561
	UA_USER_AGENT	Client user agent information	WPR 566

^a^Variable from the Clarity table: PATIENT.

^b^Variable from the Clarity table: PAT_MYC_STAT_HX.

^c^Variable from the Clarity table: MYC_PT_USER_ACCSS.

**Table 3:** Example excerpt from MyChart user access logs.

PT_MRN_ID	UA_TIME	MYC_UA_TYPE_C	UA_EXTENDED_INFO	UA_USER_AGENT	UA_SESSION_NUM
xxxxx	2018-09-17 09:20:38	Messaging	Medadvice-form	Null	56xxxx
xxxxx	2018-09-19 12:40:38	Messaging	Medadvice-form	Null	56xxxx
xxxxx	2018-09-19 12:53:12	Messaging	Medadvice-form	Null	56xxxx
xxxxx	2018-09-19 13:47:04	Login	Null	EpicMyChart-iPhone	56xxxx
xxxxx	2018-09-19 13:47:08	Provider List Widget	Get-prov-list	Null	56xxxx
xxxxx	2018-09-19 13:48:05	Messaging	Inbox message list	Null	56xxxx
xxxxx	2018-09-19 13:48:08	Messaging	Message read	Null	56xxxx
xxxxx	2018-09-19 13:48:37	Messaging	Inbox message list	Null	56xxxx
xxxxx	2018-09-19 13:49:05	Messaging	Message read	Null	56xxxx
xxxxx	2018-09-19 13:49:19	Messaging	Message read	Null	56xxxx
xxxxx	2018-09-19 13:50:07	Visits	Get future appt list	Null	56xxxx
xxxxx	2018-09-19 13:50:08	Visits	Get past appt list	Null	56xxxx
xxxxx	2018-09-19 13:50:19	Encounter Details	Past appt dat: 5xxx	Null	56xxxx
xxxxx	2018-09-19 14:06:00	Logout	Logout	Null	56xxxx

For every recorded user action, Epic’s MyChart also reports metadata that can be used to reconstruct the patient’s online behavior (ie, what was their activity and how long did it take them to accomplish that action). The variables *UA_EXTENDED_INFO* and *UA_USER_AGENT*, for example, provide important details for the categorization of user actions, necessary for quantifying the use of the different functionalities offered by the outpatient portal. The session number variable *UA_SESSION_NUM*, is instead a unique marker assigned by the server to sequences of user actions that presents patterns of consistent and continuous use.

### Data Processing

We next documented the steps taken to process the audit log file data. The result of this data processing is a dataset where each row represents a session and contains information about the time it started, its length, the device type used, and a set of frequency counts for all the activities available to the patient via the outpatient portal.

**Figure 1:**
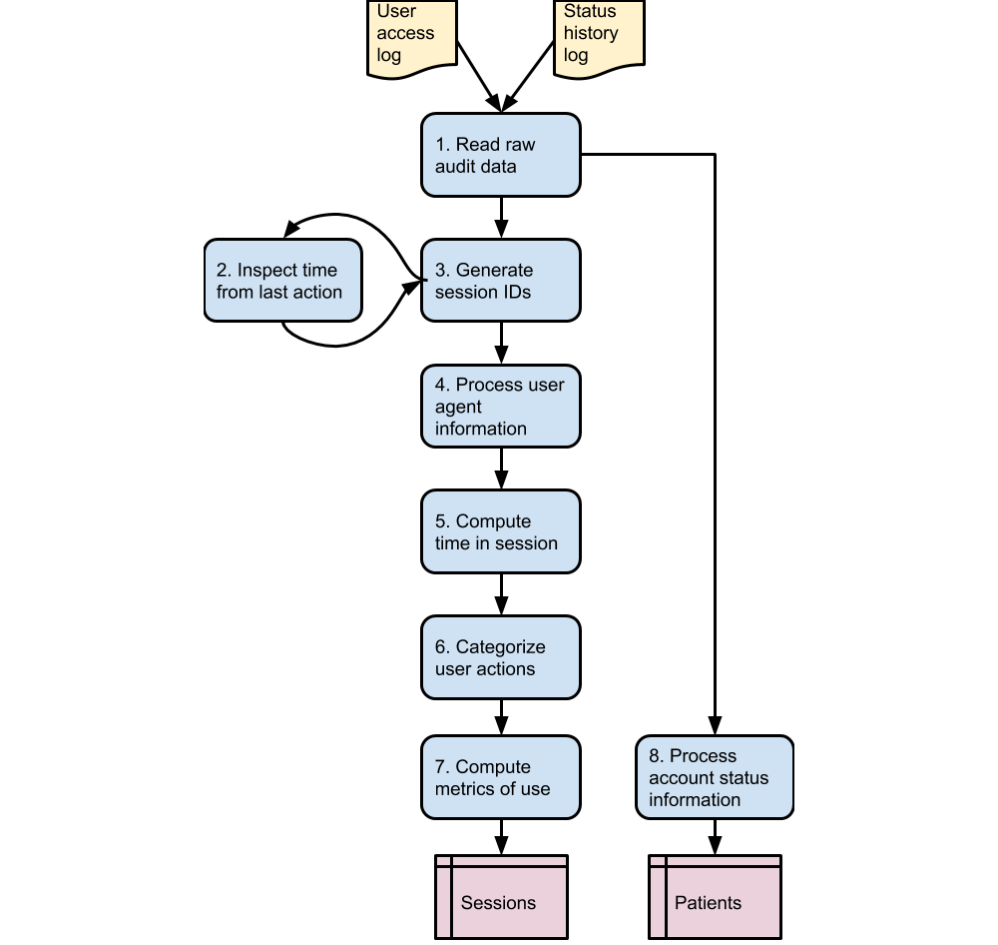
Data processing flowchart.

The steps for processing the raw data were broken down into modules, and these modules are presented in Figure 1 as a flowchart. The assumptions and specific goals of each module are described next. We have included the Stata programming code used to convert the raw data into the final data model as Multimedia Appendices 1-8.

#### Module 1: Read Raw Audit Data

In module 1, raw data are imported, and preliminary computations of secondary analytical variables are completed (see Multimedia Appendix 1). In addition to standardizing variables that contain free text or time values, this step generates a categorical variable based on the list of user actions contained in the user access log. This process both establishes MYC_UA_TYPE_C as a sorting variable and controls the ordering of concurrent user actions. For example, when multiple actions are recorded as cooccurrent, log-in attempts are listed first so as to establish a logical order of events.

#### Module 2: Inspect Time From Last Action

Module 2 quantifies and analyzes the time difference between user actions (see Multimedia Appendix 2). The intent of this step is to control and limit the time gap that identifies a coherent session.

MyChart assigns a *session number* (variable UA_SESSION_NUM) to user actions that signify continuous engagement with the outpatient portal functions; however, this variable can be unreliable. This step enables resolution of issues encountered when working with data from MyChart’s Clarity tables. The following are examples of these issues:

- Tracking of the variable might be unavailable: Institutions might deploy a patient portal but postpone tracking of the session number variable. This can result in old audit logs—even for the same patient—that have no server-assigned session information. This is true at our institution for all MyChart data older than May 15, 2016.
- The variable might be tracked intermittently: A session number might fail to be assigned to a subset of user activities, although being recorded correctly for the other activities in the enclosing sequence.
- The variable might be recorded correctly but might indicate implausible sequences of user activities: In some instances, the raw server data can indicate sessions spanning multiple days (see example in Table 3 above); this is deemed inconsistent with normal user behavior and can be resolved by enforcing a limit to the time gap between consecutive user actions.

Quantifying the time gap between consecutive actions is important as it allows the identification of implausibly long sessions and helps to correct issues with data reliability. This process occurs by calculating a parameter to limit the time gap between user actions, and it has the effect of splitting unusually long sequences into shorter ones, ensuring that the time spans recorded by the audit data measure the actual user engagement with the outpatient portal.

**Figure 2:**
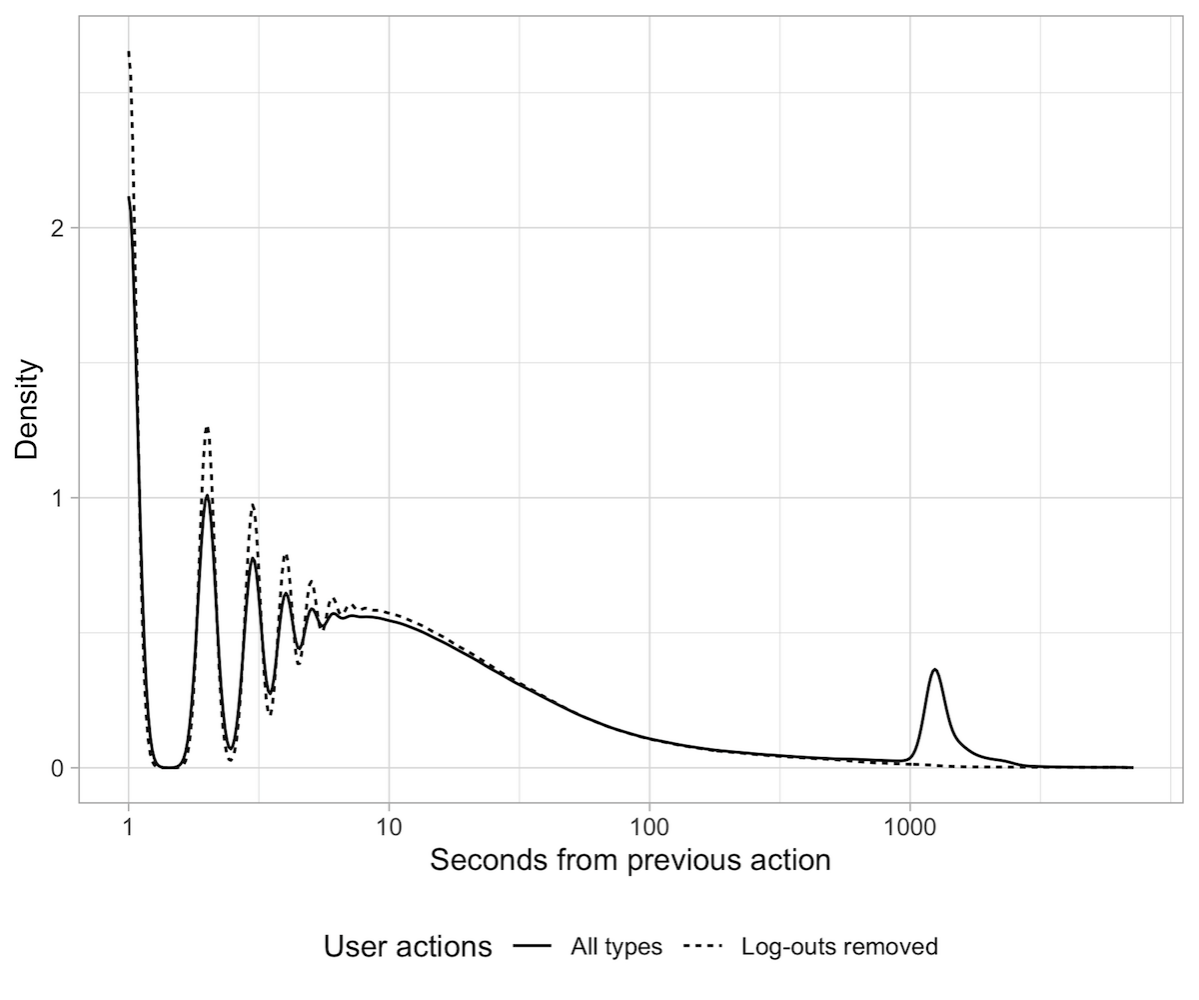
Density distribution of the time gaps between user actions.

The exact value of the parameter marking the maximum period of inactive time allowed is determined by generating a new variable, *time gap*, which stores the number of seconds separating a user action from the preceding one in the same session and analyzing its density estimation. As shown in Figure 2, this variable appears to have a wide range, with the majority of values at the lower interval, and a long right tail that is approximately log-normal, except for the spike at approximately 20 min. Upon closer inspection, this spike is explained by the time gaps associated with user actions that are recognized as *log-outs* by the MYC_UA_TYPE_C variable. The dotted line reflects the density distribution after this artifact in the data is removed.

**Figure 3:**
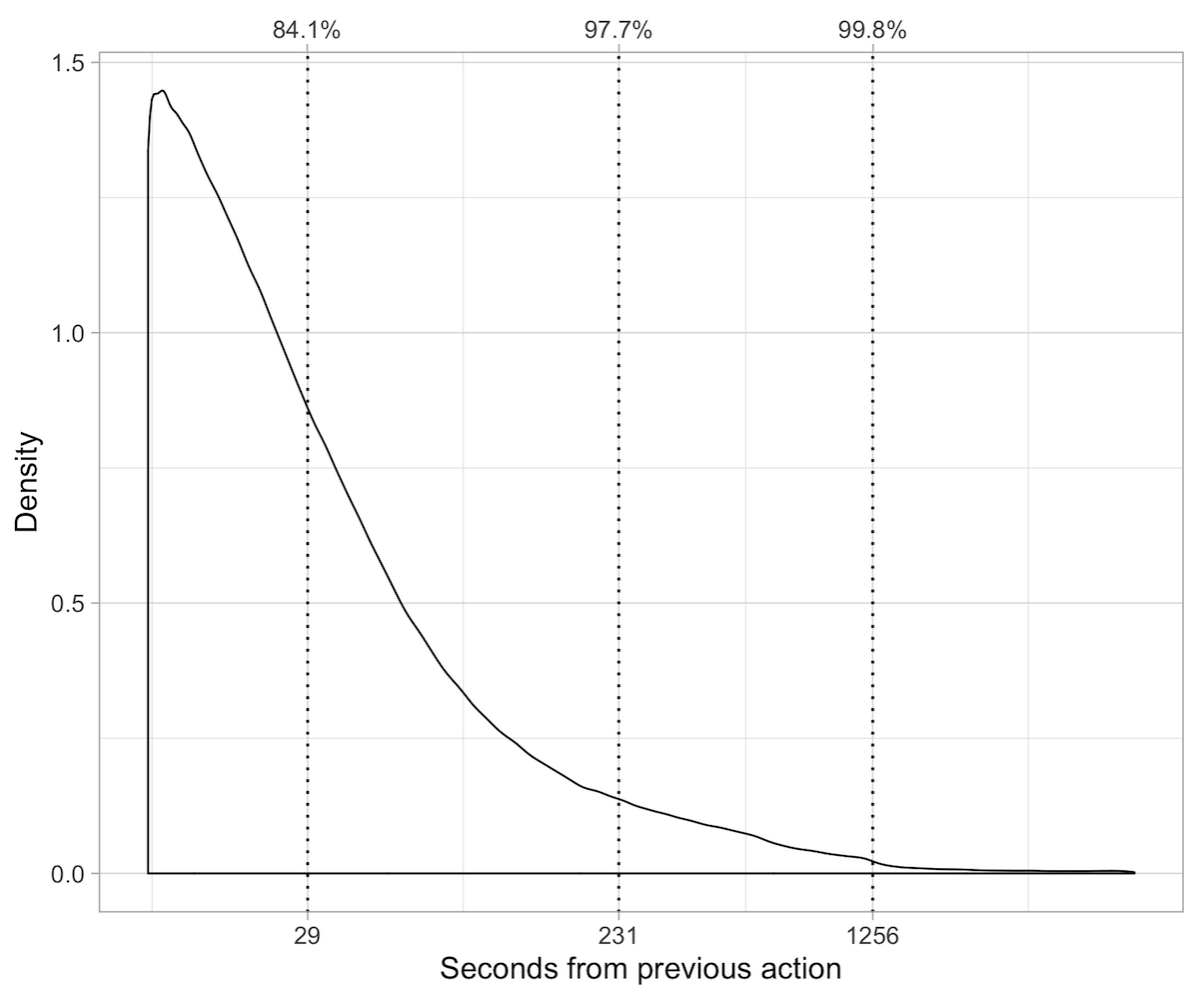
Possible thresholds for inactive time in session. Log-outs action type is removed from the dataset.

Figure 3 shows that establishing a time limit of 1256 seconds (approximately 20 min) allowed us to preserve 99.80% (3,248,044/3,254,561) of the data (excluding the *log-outs*) in their original form.

#### Module 3: Generate Session IDs

Module 3 (see Multimedia Appendix 3) creates a session ID that groups together patient activities separated by either a patient’s manual log-out or his or her failure to engage with the outpatient portal for what is deemed a significant period of time (defined in module 2). This step supersedes the system-generated UA_SESSION_NUM and groups user activities into sequences of consistent continuous use. By identifying the start and end points of the user sessions, which in most cases would match the user actions marked as *log-in*—for the start point—and as *log-out*—for the end point, an identification label is generated for every user log-in. This label is then extended to all subsequent user-recorded actions, usually up to a recorded log-out. In the event of unusual cases (ie, missing end point, start point, or both), a limit to the time gap between two consecutive user actions is enforced (calculated at approximately 20 min, as described above for module 2). Consequently, all sequences of user actions that are recorded after an inactive time greater than the calculated threshold are regrouped, and a newly created session identification label is generated.

#### Module 4: Process User Agent Information

User agent information is used in this module to infer the type of device the patient uses to access the outpatient portal (see Mutimedia Appendix 4). Metadata associated with the log-in attempts in the UA_USER_AGENT and UA_EXTENDED_INFO variables can consistently indicate whether access is originating from a desktop or mobile platform. As part of this step, the device type information is stored in a newly created variable, *se_device*, that marks all the actions belonging to the same session.

#### Module 5: Compute Time in Session

Module 5 calculates the time taken in seconds for each sequence of actions marked with the same session ID (see Multimedia Appendix 5). The algorithm excludes all the *log-outs* from the computation to return an effective time in session. As shown in Figure 3, log-outs consistently overestimate the time a patient remains engaged with the outpatient portal; therefore, we filter out log-outs to avoid artifactual results (see Results section).

#### Module 6: Categorize User Actions

This module classifies user actions under the categories of the outpatient portal’s functions (see Multimedia Appendix 6). The list of labels used to classify user action types, together with their associated grouping functions, are reported in Table 4. This list has been compiled after extensive testing involving the following:

- Controlled interaction with the functionalities of the outpatient portal in a test environment using quality assurance automation tools to verify the traces left by user actions on the server side: As a single user action can be instantiated via multiple system calls—some essential and others ancillary to many other calls, with each one independently leaving traces of its own activation in the audit log—there is no one-to-one correspondence between the user behavior on the client side and the logged portal functions on the server side. We, therefore, undertook a systematic and exhaustive exploration of the functionalities made available to patients via MyChart in a test environment. This test environment mirrored the production portal and included the availability of Clarity tables and automation tools such as Selenium [18], a tool common in quality assurance testing that allows for the reproducibility of workflows and fine-grained control over the timing of actions. This test environment allowed us to generate reproducible patterns in the log data and required the extraction of the signals associated with specific user activities.
- Investigation of more complex cases with information technology (IT) specialists at OSUWMC: In cases where the sole user behavior was not enough to produce unambiguous logs, the help of an IT specialist was enlisted to produce the intended response from the server or to observe in real time if and when fields in the Clarity tables were populated, as a means to verify that the correct data were being collected.
- Clarification of the implementation details with technical support from Epic: In rare cases, information related to implementation details and other clarifications were obtained directly from Epic through their technical support services.
  The labels of the action types and portal functions offer a 2-level taxonomy for the categorization of user actions that employ terms from the MyChart user interface. At the higher level, the portal functions are named as follows:
  - Messaging: Contains links to the message center, letters to the patient, and an option to request prescription refills
  - Visits: List of past and upcoming visits and the ability to schedule and cancel appointments
  - My record: List of medications, allergies, medical history and immunization status, health summary and test results, preventive care, and a summary table of the plan of care
  - Medical tools: Sharing medical records with others or with other services (Lucy and MyChart Central), participate in research studies, and connect tracking devices (eg, Fitbit)
  - Billing: Bills, insurance information, and estimates for common procedures
  - Resources: Frequenty asked questions, terms and conditions, and patient education
  - Proxy: Request proxy status or renew proxy requests
  - Preferences: Personal settings, security settings, and notification preferences
  Auxiliary action types, such as log-ins, log-outs, and 2-factor authentication, were flagged with a *Miscellaneous* label, but were otherwise excluded from the calculation of portal use metrics. Frequency counts associated with the *Resources* portal function are absent because this set of action types contains links to patient educational information that are all pointers to resources hosted outside MyChart; interaction with these elements, if present, is not captured before the patients navigate away from MyChart.

**Table 4:** Labels for action types and portal functions used to categorize raw audit log data from Epic's MyChart. For each label, we report the count of sessions in which the action was performed and the percentage represented by the session count over the total number of sessions (N=451,762).

Action type	Portal function	Sessions, n (%)
Letters	Messaging	5318 (1.18)
Message center	Messaging	146,394 (32.41)
Send new message	Messaging	45,391 (10.05)
Appointment details	Visits	17,393 (3.85)
Cancel an appointment	Visits	3665 (0.81)
Driving directions	Visits	232 (0.05)
eCheck-in^a^	Visits	25,246 (5.59)
Schedule an appointment	Visits	67,039 (14.84)
Telemedicine	Visits	13 (0.00)
Upcoming tests	Visits	6784 (1.50)
Allergies	My record	24,345 (5.39)
Current health issues	My record	18,304 (4.05)
Flowsheet	My record	184 (0.04)
Health summary	My record	8989 (1.99)
Immunizations	My record	14,273 (3.16)
Medications	My record	14,450 (3.20)
My conditions	My record	2009 (0.44)
Preventive care	My record	4854 (1.07)
Test results	My record	10,6043 (23.47)
Consolidate EMR^b^	Medical tools	3178 (0.70)
Download my record	Medical tools	53 (0.01)
Research studies	Medical tools	392 (0.09)
Share my record	Medical tools	62 (0.01)
Wallet card	Medical tools	687 (0.15)
Who accessed my record	Medical tools	1267 (0.28)
Bill payment	Billing	6862 (1.52)
Billing account details	Billing	0 (0.00)
Billing account summary	Billing	11432 (2.53)
Change paperless status	Billing	111 (0.02)
Estimates	Billing	838 (0.19)
Insurance summary	Billing	15,538 (3.44)
Update insurance	Billing	11,401 (2.52)
Patient education	Resources	0 (0.00)
Terms and conditions	Resources	0 (0.00)
Proxy forms	Proxy	5763 (1.28)
Proxy renewal request	Proxy	0 (0.00)
Request child proxy access	Proxy	0 (0.00)
Request proxy access	Proxy	84 (0.02)
Switch proxy context	Proxy	556 (0.12)
About me	Preferences	0 (0.00)
Manage my accounts	Preferences	0 (0.00)
Notifications	Preferences	4349 (0.96)
Personalize	Preferences	1768 (0.39)
Security settings	Preferences	4543 (1.01)

^a^eCheck-in: Electronic check-in.

^b^EMR: electronic medical record.

#### Module 7: Compute Metrics of Use

The goal of this module is to calculate count values for the portal usage metrics and merge them with the other session-level variables (see Multimedia Appendix 7). The output at the end of this step is a dataset ready for data analysis, organized at the session level. For each constructed session, information about the device used and the time spent in sessions are reported along with frequency counters for the MyChart activities of the taxonomy developed during the previous step. In addition, a reference to the MRN enables the computation of statistics at the patient level as well as the merging of demographic information, when it is available.

Before computing usage metrics, the module filters out redundant records of the same user action from the log file data. Owing to the implementation details affecting users accessing MyChart from a mobile platform, the audit reports can contain many duplicate rows as the same user action can be logged at intervals of 2 seconds until the patient navigates away from the page that is triggering the behavior. Although this does not affect the time in session, it results in inflated action counts for patients accessing the outpatient portal via a mobile phone.

The first part of the code for module 7 controls for the presence of a series of identical user actions, with matching values in both the MYC_UA_TYPE_C and UA_EXTENDED_INFO variables, and deletes all but the first occurrence in each series.

#### Module 8: Process Account Status Information

The account status information is processed in this module to create a dataset organized at the patient level (see Multimedia Appendix 8). The module accepts as input the list of status changes recorded for each patient’s account and processes it to determine the time the patient’s account was activated and the current account status. The status of a MyChart account can vary over time, marking, among other things, the sign-up phase (status: *pending activation*), a phase of regular use (status: *active*), and potentially the termination of online use (status: *inactivated*). The resulting dataset, with one row per patient, is suitable to be merged with demographic data to support more detailed analysis of the outpatient portal use.

## Results

### Session Counts and Activation Status

Using this protocol, we applied the processing steps outlined above to the log files of MyChart activity for the patients in our sample. The following sections present and summarize the results of our analysis of metrics pertaining to the frequency and comprehensiveness of outpatient portal use.

All descriptive statistics were calculated using data from sessions with nonzero duration and from patients who had at least two valid sessions on record. As a result, we discarded all the log-in attempts that did not result in actual user navigation of the outpatient portal; we further refined the dataset to exclude patients who never engaged with MyChart beyond the time required to register an account.

**Figure 4:**
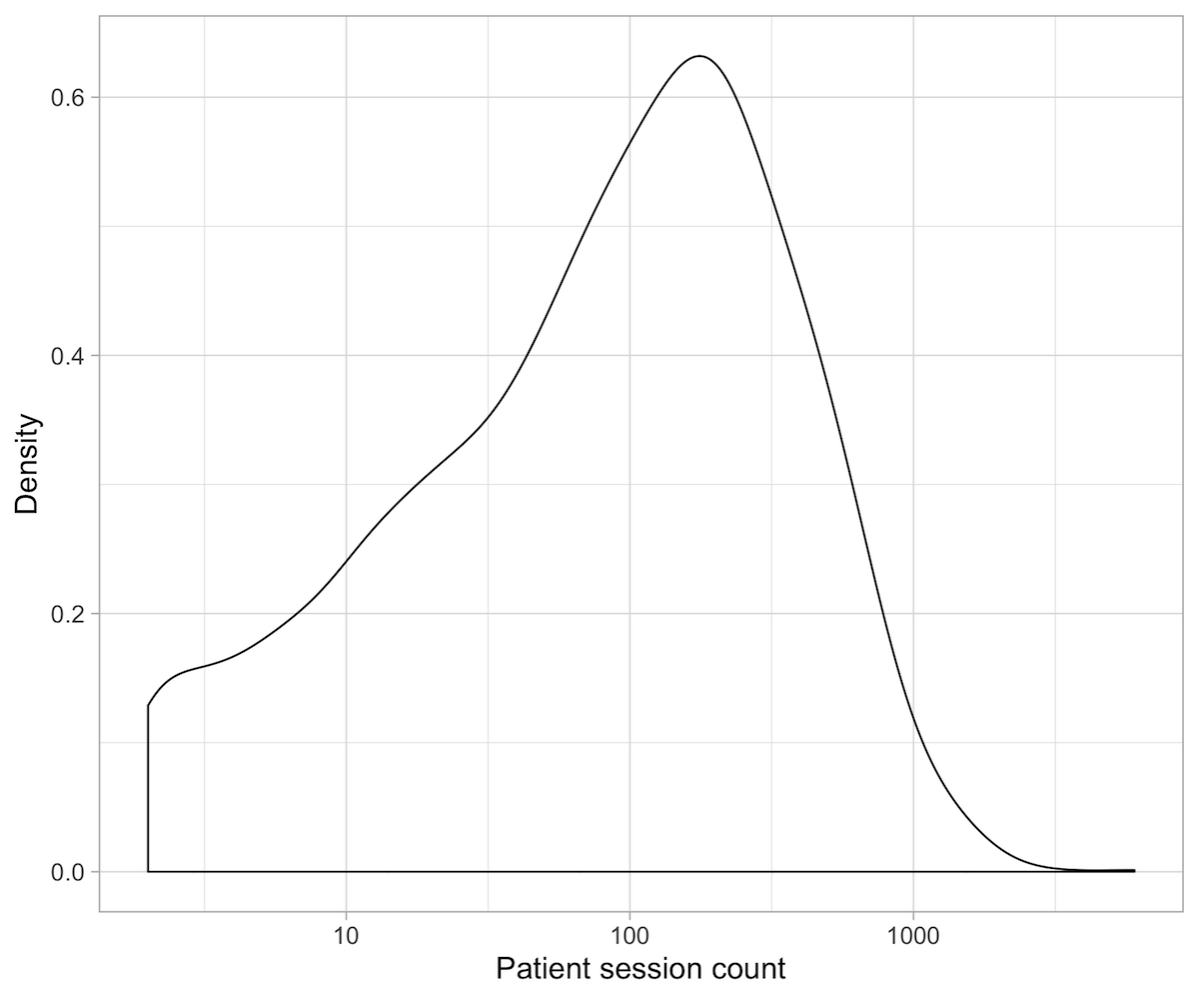
Distribution of session counts per patient.

Overall, the dataset contained 451,762 valid sessions (total number of sessions=482,443) for 2511 active patients (total number of patients=2607). On average, each patient had 180 sessions on record; the median number of sessions per patient was 94 sessions, with an IQR of 207 and a maximum recorded count of 6012 (Figure 4).

MyChart account status of the patients included in the final dataset is reported in Table 5, together with the account status of the general hospital population. Not all patient accounts were flagged as *activated*. The majority of *inactivated* accounts were accounts that had been previously active but had been closed because of the death of their owner (*patient deceased*, n=125). The remaining cases represented individuals who never formally concluded the sign-up process or inadvertently invalidated their status after activating their account, but nonetheless engaged with the outpatient portal; as a result, their audit data had been consistently recorded.

The distribution of patients with activated MyChart accounts across age groups (Table 6), although inversely related to the ages of patients, shows that activated accounts remain the largest category across our sample population.

**Table 5:** Patients’ MyChart account status for sampled and overall hospital population.

Account status	Sample population (N=2511), n (%)	Hospital population (N=70,076), n (%)
Activated	2360 (93.99)	39,373 (56.18)
Inactivated	134 (5.34)	12,678 (18.09)
Patient declined	4 (0.16)	6433 (9.18)
Pending activation	13 (0.52)	11,592 (16.54)

**Table 6:** MyChart account status as a function of patient age group.

Age group (years)	Account status (N=2511), n (%)				
	Activated	Inactivated	Patient declined	Pending activation	Total
18-29	357 (15.13)	7 (5.22)	0 (0.00)	4 (30.77)	368 (14.66)
30-39	468 (19.83)	14 (10.45)	0 (0.00)	2 (15.38)	484 (19.28)
40-49	456 (19.32)	21 (15.67)	1 (25.00)	0 (0.00)	478 (19.04)
50-59	500 (21.19)	36 (26.87)	1 (25.00)	1 (7.69)	538 (21.43)
60-69	429 (18.18)	38 (28.36)	1 (25.00)	3 (23.08)	471 (18.76)
70+	150 (6.36)	18 (13.43)	1 (25.00)	3 (23.08)	172 (6.85)

### Frequency of Use

**Figure 5:**
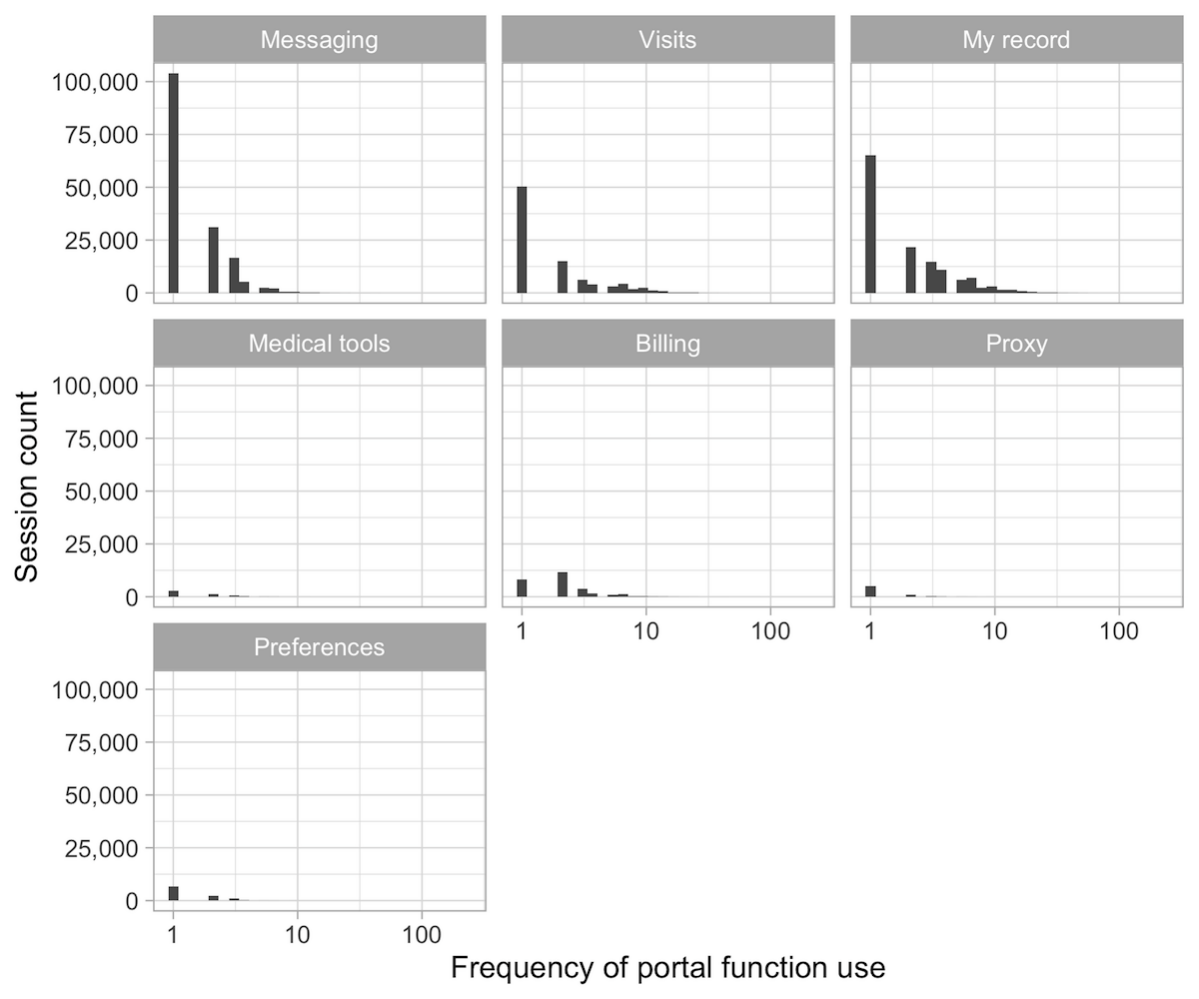
Frequency distribution of the use of the different portal functions across all the sessions in the dataset.

**Figure 6:**
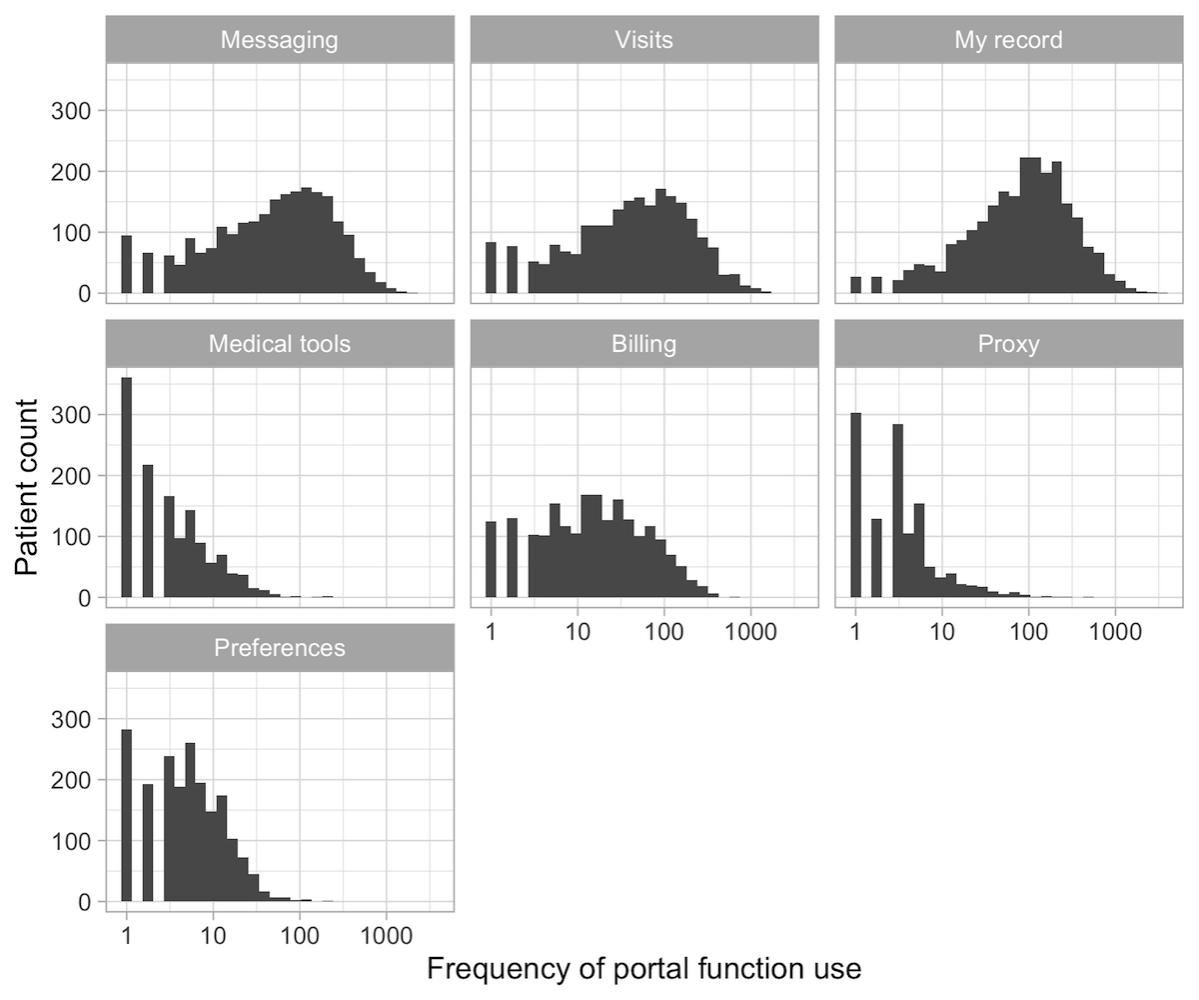
Frequency distribution of the use of the different portal functions across the patient population.

The results in Table 7 show descriptive statistics for the frequency counts associated with each MyChart portal function as defined by the taxonomy of user actions (see also Figures 5 and 6). Of note, at the session level, the median frequency count for the use of all MyChart functions is 0. Although in most cases the ranges are quite broad, the 0 medians suggest that the values are concentrated at lower intervals.

**Table 7:** Frequency of use at session and patient levels for each of the MyChart portal functions.

Portal function^a^	Session level (N=451,762)					Patient level (N=2511)			
	Minimum value	Median	Maximum value	IQR	Minimum value		Median	Maximum value	IQR
Messaging	0	0	84	1	0		51	2087	134
Visits	0	0	113	0	0		38	1670	108
My record	0	0	227	1	0		87	3503	171
Medical tools	0	0	12	0	0		1	223	3
Billing	0	0	81	0	0		10	659	31
Proxy	0	0	9	0	0		0	498	3
Preferences	0	0	15	0	0		3	228	7

^a^Frequency counts associated with the *Resources* function on the outpatient portal are absent because of technical reasons (see module 6 in the Methods section).

### Comprehensiveness of Use

Comprehensiveness of portal function use, defined according to our proposed data model as the number of distinct functions accessed by the patient, provides a summary of how the patient engaged with the outpatient portal.

Comprehensiveness of use results are presented in Table 8. At the session level, in most cases, patients were found to engage with the portal for one specific function; only 17.45% (78,787/451,762) of the sessions were linked to activities involving more than one portal function. At the patient level, most individuals used 3 to 4 of the portal’s functions over the course of the study.

**Table 8:** Comprehensiveness of use.

Counts of portal function used	Session (N=451,762), n (%)	Patient (N=2511), n (%)
0	117,819 (26.08)	14 (0.56)
1	255,156 (56.48)	64 (2.55)
2	57,761 (12.79)	274 (10.91)
3	16,215 (3.59)	696 (27.72)
4	4271 (0.95)	1066 (42.45)
5	491 (0.11)	349 (13.90)
6	47 (0.01)	46 (1.83)
7	2 (0.00)	2 (0.08)

### Artifacts in the Data

**Figure 7:**
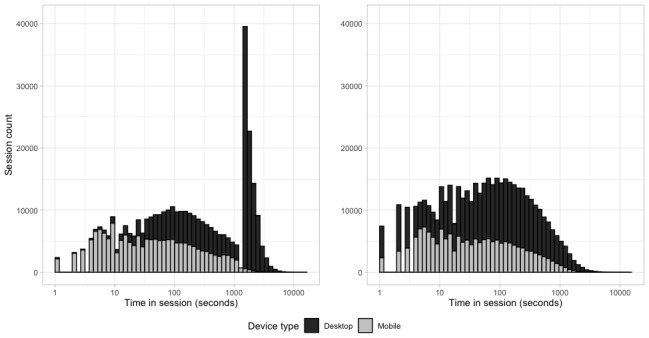
Distribution of time in session by device type. On the left, time in session is calculated on raw server data. On the right, time in session is calculated after processing user access logs, removing log-outs action type, and enforcing a limit of 1256 seconds to the time patients can be idle on the portal.

Of note, our data processing methodology also uncovered a series of artifacts in the data, which we were able to correct. The first artifact affected the measurement of the time patients actively engaged with the portal. The distribution of the time spent in sessions calculated on raw server logs showed a bimodal distribution, with most sessions registering a length of a few minutes and a sizable group of longer sessions of approximately 20 min to 22 min (see graph of the left in Figure 7). This second group exclusively comprised patients connecting via a desktop computer (as opposed to a mobile phone), whose connections were kept alive by the server despite the patient being idle on the portal (see description of module 2 in the Methods section). The same analysis performed on audit logs processed with our proposed methodology showed how the distribution of the length of the sessions is instead approximately log-normal (see the graph on the right in Figure 7) and, by getting rid of idle time, better approximated the actual engagement duration of the patient with the portal.

A second type of artifact involved the audit logs mined from the mobile client. As documented in module 7 of the Methods section, duplicate data for MyChart activities recorded during mobile sessions can affect the frequency of use metrics if not properly identified and filtered.

## Discussion

### Principal Findings

Patient portals have the potential to improve patient engagement and health outcomes. Our intention with this preliminary study was to uncover the main artifacts in the data and to build a model that starts a conversation on how to use outpatient portal log files for research purposes. As previously noted, evaluations of portal use have, to date, mainly been conducted through the analysis of self-reported behaviors [8]. Furthermore, a review conducted by Irizarry et al [19] about patient portals and patient engagement highlighted the lack of objective measures for usability testing and attributed this gap to the high costs of this type of analysis in terms of time and effort. Our analysis of log files to develop metrics responds to this problem and presents the opportunity to augment self-reports of portal use with the new behavioral measure for portal function use we have described.

Data analytics research has additionally proven effective in describing portal adoption for targeted subpopulations [20]. Our methodology demonstrates how to incorporate information about the status of patient portal accounts in the final dataset (see script for module 8 in the Multimedia Appendix 8). This approach provides information grounded in longitudinal changes to patient accounts that can provide the basis for evaluating portal adoption and use and inform targeted strategies for implementation, improvement, and optimization of use.

Following the recommendations of Sieverink et al [16], our approach highlights the importance of examining outpatient portal use based on how the tool was actually used by patients (ie, including the different functions or elements within it) as opposed to simply reporting the number of log-ins and the frequency of use of the tool. Our results also demonstrate that, based on the level of analysis (ie, patient or session), the patterns of outpatient portal use by function show variability. Interestingly, this variability is more pronounced at the patient level as opposed to the session level. In contrast, at the session level, the usage pattern showed frequent but highly specialized interactions: patients visit the outpatient portal to achieve a single goal and navigate away from it as soon as the goal is achieved. Considering use metrics at the patient level instead, we found that although the portal use spread across all functions, many patients engaged only with a subset of those—a phenomenon that was also detected by Huerta et al [17] in their log file assessment of inpatient portal use.

Although the scope of this descriptive study is limited to the presentation of usage metrics constructed from data elements presently available in our institution’s outpatient portal database, the integration of these data with other data sources will present additional opportunities for analysis. Future research on outpatient portal use can explore potential confounders that may influence use, such as demographics, diagnoses, treatments, and patients’ communications with care team members. In addition, further work will permit the identification and characterization of user clusters, similar to the work of Jones et al [15] on outpatient portal use among chronic patients and of Fareed et al [21] on inpatient portal use; this approach will improve our understanding of the observed differences in usage patterns at the patient level.

### Limitations

We acknowledge two main limitations to this study. First, although we have attempted to generalize our methodology and note procedures that are relevant to log file analysis independent of the institutional context, our study was limited to the analysis of log files from a single outpatient portal system that had retained many of the vendor’s technical specifications. For instance, we were unable to obtain log files for functions connected to health educational resources because of the decisions by the EHR vendor and/or our institution to not track such actions. As such information is valuable in measuring patient engagement with their health, additional study is warranted in contexts where these data are collected. Our choice to document the processing steps for our data analysis in the form of Stata do files was motivated by the consideration that enabling reproducible research might bridge the institutional gap and mitigate this limitation. However, applying our methodology in other contexts or with other portal systems will require accounting for the institutional idiosyncrasies and contingencies that influence how the log files are parsed.

Second, with regard to identifying a level of analysis for portal use, as we were working with outpatient records, our data did not have a well-defined cutoff period. This is different from the case of inpatient portal data, where the hospital admission period provides a discrete time frame and level of analysis. We believe that with outpatient portal use, linking portal use to patients’ medical history (eg, outpatient portal use during a specific index event such as major surgery) could guide researchers toward more meaningful ways to aggregate log file data and we aim to pursue such an approach in the future.

### Conclusions

Although there has been rapid adoption of patient portals across health care organizations, traditional approaches to assessing their impact have not leveraged the potential of detailed audit log file analysis as we have presented. We aimed to model an approach to the outpatient log file analysis by presenting our methodology and describing the challenges associated with accessing and analyzing this type of data. Improving understanding of this process should enable researchers and practitioners to consider this methodology in future studies of outpatient portal use and impact.

## Appendix

Multimedia Appendix 1 Stata module 1: read raw audit data.

Multimedia Appendix 2 Stata module 2: inspect time from last action.

Multimedia Appendix 3 Stata module 3: generate session ids.

Multimedia Appendix 4 Stata module 4: process user agent information.

Multimedia Appendix 5 Stata module 5: compute time in session.

Multimedia Appendix 6 Stata module 6: categorize user actions.

Multimedia Appendix 7 Stata module 7: compute metrics of use.

Multimedia Appendix 8 Stata module 8: process account status information.

## Abbreviations

EHR: electronic health record
IT: information technology
MRN: medical record number
MU: Meaningful Use
OSUWMC: Ohio State University Wexner Medical Center
